# Emergence and clonal expansion of *Aeromonas hydrophila* ST1172 that simultaneously produces MOX-13 and OXA-724

**DOI:** 10.1186/s13756-023-01339-4

**Published:** 2024-03-03

**Authors:** Xinfei Chen, Minya Lu, Yao Wang, Han Zhang, Xinmiao Jia, Peiyao Jia, Wenhang Yang, Jiawei Chen, Guobin Song, Jianguo Zhang, Yingchun Xu

**Affiliations:** 1 Department of Laboratory Medicine, State Key Laboratory of Complex Severe and Rare Diseases, Peking Union Medical College Hospital, Chinese Academy of Medical Science and Peking Union Medical College, Beijing, China; 2 Beijing Key Laboratory for Mechanisms Research and Precision Diagnosis of Invasive Fungal Diseases (BZ0447), Beijing, China; 3 Medical Research Center, Peking Union Medical College Hospital, Chinese Academy of Medical Science & Peking Union Medical College, Beijing, China; 4 Graduate School, Chinese Academy of Medical Science and Peking Union Medical College, Beijing, China; 5 Department of Clinical Laboratory, The Second Affiliated Hospital of Nanchang University, Nanchang, China; 6 Department of orthopaedic, Peking Union Medical College Hospital, Chinese Academy of Medical Science and Peking Union Medical College, Beijing, China

**Keywords:** *Aeromonas hydrophila*, ST1172, Carbapenem resistance

## Abstract

**Background:**

*Aeromonas hydrophila* infections can cause gastrointestinal symptoms such as diarrhea; however, deep infections are rarely reported. Outbreaks of *A. hydrophila* are reported more frequently in fish, poultry, and snakes than in humans. This study aimed to track clonal relatedness of deep infections caused by *A. hydrophila* using whole genome sequencing (WGS).

**Methods:**

We collected three isolates of *A. hydrophila* in July 19 to August 29, 2019, from patients that underwent spine surgery. Accurate species identification was performed using whole-genome average nucleotide identity (ANI). Antimicrobial susceptibility testing was performed using a VITEK 2 automated AST-N334 Gram-negative susceptibility card system. Antimicrobial resistance and virulence genes were identified using the Comprehensive Antibiotic Resistance Database and Virulence Factor Database VFanalyzer.

**Results:**

All three isolates were identified as *A. hydrophila* based on ANI and multilocus sequence typing analysis revealed that *A. hydrophila* belonged to a novel sequence type (ST1172). All three isolates were susceptible to amikacin and levofloxacin; however, they were resistant to piperacillin/tazobactam, ceftriaxone, cefuroxime, cefoxitin, and imipenem. Isolate 19W05620 (patient 3) showed increased ceftazidime resistance (minimum inhibitory concentration ≥ 64 µg/mL). All three isolates possessed the same chromosomally encoded β-lactamases, including *bla*_OXA-724_ (β-lactamase), *imiH* (metallo-β-lactamase), and *bla*_MOX-13_ (AmpC) in plasmids.

**Conclusions:**

Our study validated the transmission of a novel carbapenem-resistant *A. hydrophila* sequence type (ST1172) in patients that underwent spine surgery. Control measures should be developed to prevent dissemination of *A. hydrophila* in the hospital setting.

**Supplementary Information:**

The online version contains supplementary material available at 10.1186/s13756-023-01339-4.

## Background

*Aeromonas hydrophila* is a gram-negative rod-shaped bacteria that possesses polar flagella and occurs ubiquitously in aquatic environments [1]. As a foodborne pathogen, *A. hydrophila* often causes gastrointestinal disease in humans, but can also cause extraintestinal infections including necrotizing fasciitis and sepsis [2]. *A. hydrophila* infections are widespread, with reported outbreaks in farm-raised snakes and in-hospital transmission [3, 4]. There have also been reports of nosocomial infections caused by various carbapenemase-producing strains of *Aeromonas* at the UCLA Medical Center in the United States [5].

The ongoing emergence of multi-drug resistant strains has raised concerns. As a species with intrinsic and acquired resistance, *A. hydrophila* shows a decreasing susceptibility to antimicrobial drugs. Intrinsic resistance in *A. hydrophila* is conferred by chromosomally encoded β-lactamases such as Ambler class C (AmpC), while acquired resistance is transmitted predominantly via resistance plasmids [6]. Notably, extended-spectrum-β-lactamase (ESBL)-producing *A. hydrophila* strains have been isolated from clinical specimens [6]. The emergence of highly virulent strains represents a serious problem for the farming industry; for example, the highly virulent *A. hydrophila* ST251 was reported to cause motile *Aeromonas* septicemia in fish [7]. Studies on the virulence genes of *A. hydrophila* revealed multifactorial virulence factors that include adhesins (type IV pilus, MSHA type IV pili, tap type IV pili, and type I pili), motility factors (polar flagella), secretion systems (exe T2SS and T6SS), and toxins (aerolysin and RtxA).

Deep *A. hydrophila* infections are rarely reported and little research has been done on the associated clinical prognosis. In this study, we isolated carbapenemase-producing *A. hydrophila* from four patients with post-surgical infections in the same ward of the orthopedic department from July to September, 2019. We performed molecular typing of the clinical isolates to evaluate the possible occurrence of an outbreak, and resistance genes were evaluated to determine the resistance mechanisms.

## Methods

### Isolates and antifungal susceptibility testing

Only the first *A*. *hydrophila* isolate from patient 1, 3, and 4 was included in the study, while the strains isolated from patient 2 could not be revived from long-term storage. Strain 19B23009 (patient 1) was isolated from a blood culture, while strain 19W05620 and 19W06265 (patient 3, 4) were isolated from drainage fluid. Three isolates were identified at the species level using an Autof-MS 1000 system (Autobio, Zhengzhou, China). SpeciesFinder 2.0 was used to identify the three isolates based on 16s rRNA sequence [8]. Antimicrobial susceptibility testing was performed using a VITEK 2 automated AST-N334 Gram Negative susceptibility card system (bioMérieux, Marcy-l’Étoile, France). Susceptibility to meropenem (Oxoid, Basingstoke, UK) was tested using a Kirby-Bauer disk. The in vitro clinical breakpoints of antimicrobial agents against *A. hydrophila* were based on the Clinical and Laboratory Standards Institute guidelines for 14 different antimicrobial agents [9].

### Settings

Microbe samples were collected from all disinfectants and sterile cotton swabs utilized in the orthopedic department of Peking Union Medical College Hospital, as well as any potentially contaminated bone grain, surgical instruments, and infusion packs used in the surgical room between June 26 and September 3, 2019. Environmental surface sampling was conducted as a surrogate for actual air sampling within the orthopedic department and surgical room. Fecal cultures were conducted for all four patients to detect *A. hydrophila* infections.

### DNA extraction and whole-genome sequencing

Genomic DNA was extracted from *A. hydrophila* isolates as previously described [10]. The DNA library was constructed using NEBNext® Ultra™ (New England Biolabs, Ipswich, MA, USA) following the manufacturer’s instructions. An Agilent 2100 Bioanalyzer was used for quality confirmation. Genome sequencing was performed using an Illumina NovaSeq 6000 at Beijing Novogene Bioinformatics Technology (Beijing, China). The raw data were deposited in the NCBI BioProject database (https://www.ncbi.nlm.nih.gov/bioproject/; BioProject accession number: PRJNA912376).

### Comparative genomic analysis and core-genome alignment

Paired-end sequences with > 100× coverage were used for the bioinformatics analysis. Scaffolds were assembled using SPAdes 3.13.1 [11] and Prokka 1.12 for annotation [12]. The total scaffold number and N50 scaffold size of the genome assemblies were calculated using TBtools-II 2.012 [13]. The draft annotated genomes were visualized using Proksee [14]. Taxonomic affiliation was determined using the average nucleotide identity (ANI) based on BLAST+ (ANIb) and the Tetra-nucleotide signature correlation index using the JSpecies Web Server (JSpace WS) with default parameters [15]. Comparative genomic analysis was performed using the genome sequences of *A. allosaccharophila* CECT4199, *A. aquatica* AE235, *A. bestiarum* CECT 4227, *A. bivalvium* CECT 7113, *A. caviae* CECT 838, *A. dhakensis* CIP 107,500, *A. encheleia* CECT 4342, *A. enteropelogenes* CECT 4487, *A. eucrenophila* CECT 4224, *A. finlandensis* 4287D, *A. fluvialis* LMG 24,681, *A. hydrophila* ATCC 7966, *A. hydrophila* subsp. *ranae* CIP 107,985, *A. jandaei* CECT 4228, *A. lacus* AE122, *A. lusitana* MDC 2473, *A. media* CECT 4232, *A. piscicola* LMG 24,783, *A. popoffii* CIP 105,493, *A. salmonicida* subsp. *salmonicida* ATCC 33,658, *A. sanarellii* LMG 24,682, *A. simiae* CIP 107,798, *A. taiwanensis* LMG 24,683, *A. tecta* CECT 7082, and *A. veronii bv. veronii* CECT 4257 using Roary 3.11.2 [16]. A phylogenetic tree was constructed using maximum likelihood method implemented in RAxML with 1000 bootstrap replicates to investigate the genetic relationships of *A. hydrophila* [17].

### Multilocus sequence typing of isolates

Six housekeeping genes (DNA gyrase B [*gyrB*], chaperonin groEL [*groL*], citrate synthase [*gltA*], methionine tRNA ligase [*metG*], phenolphthiocerol/phthiocerol polyketide synthase subunit A [*ppsA*], and RecA [*recA*]) extracted from the draft genomes were selected for the multilocus sequence typing (MLST) analysis. Housekeeping gene sequences were uploaded to PubMLST (https://pubmlst.org/bigsdb?db=pubmlst_aeromonas_seqdef&page=sequenceQuery) and the sequence type (ST) of the three isolates was determined by matching them with publicly available data. For the phylogenetic analysis, 51 allele sequences were exported from PubMLST (https://pubmlst.org/bigsdb?db=pubmlst_aeromonas_seqdef&page=plugin&name=SequenceExport&scheme_id=1), and the STs were aligned and analyzed using MEGA X via the maximum likelihood method [18].

### Detection of virulence and drug-resistance genes

Antimicrobial-resistance genes were identified using the Comprehensive Antibiotic Resistance Database (CARD; https://card.mcmaster.ca/). Virulence genes were screened using the Virulence Factor Database (VFDB; www.mgc.ac.cn/VFs). Briefly, VFanalyzer (www.mgc.ac.cn/cgi-bin/VFs/v5/main.cgi?func=VFanalyzer) was used to automatically perform a systematic screening of known/potential virulence factors in the given complete/draft bacterial genomes.

## Results

### Clinical history

**Fig. 1:**
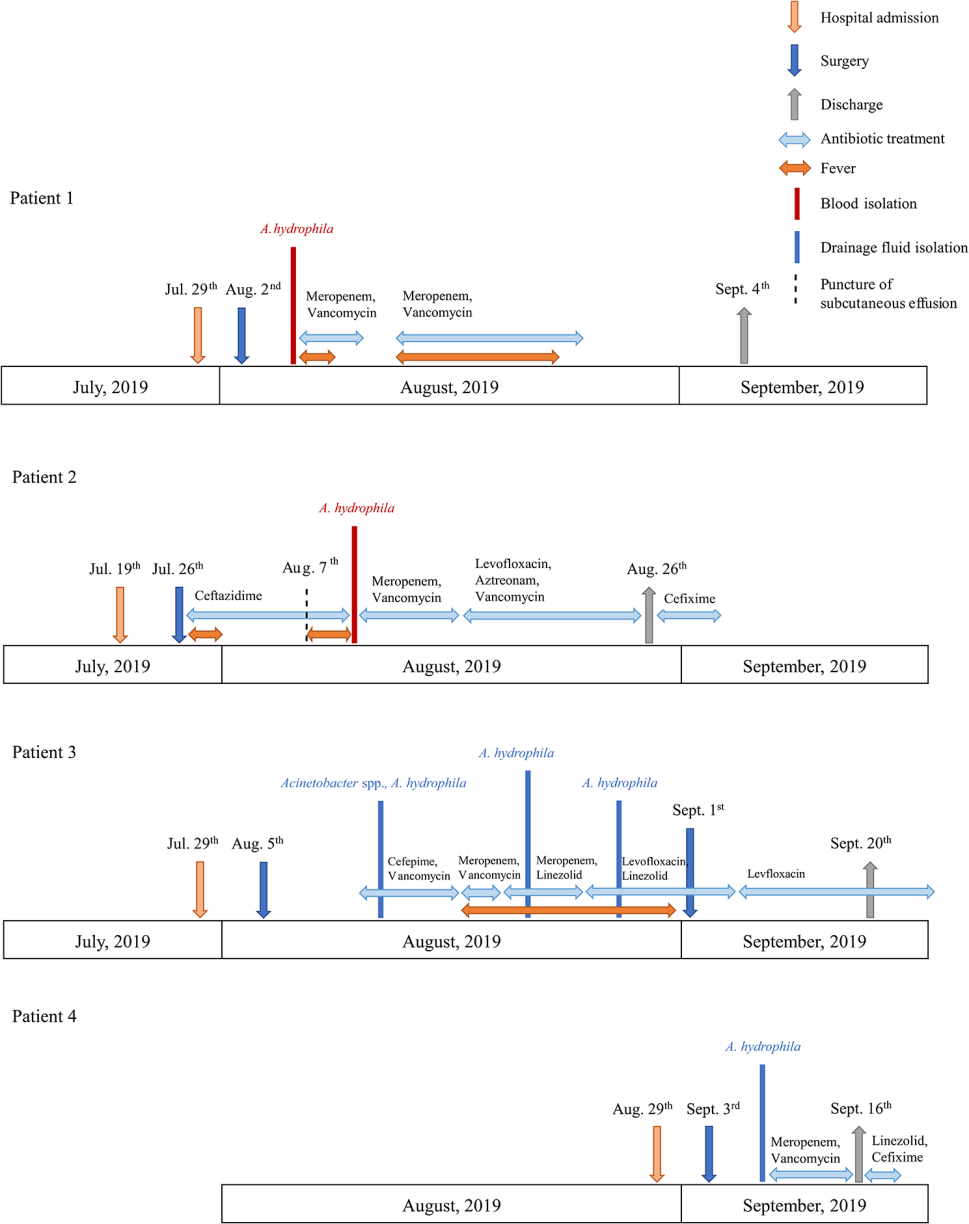
Epidemiology of the *A. hydrophila* outbreak. Colored text and bars represent the isolate sources

**Fig. 2:**
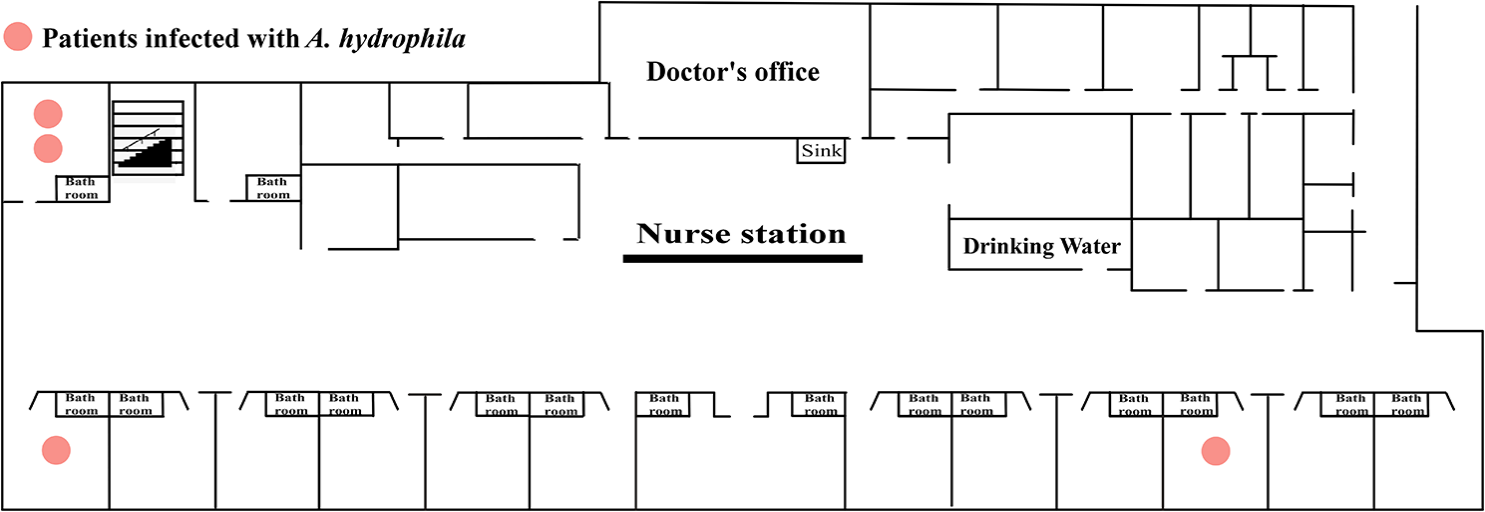
Locations of patients infected with *A. hydrophila* in the hospital

Four patients aged 36–77 years were admitted to the orthopedic ward of Peking Union Medical College Hospital between July and August, 2019, and presented with cervical spondylosis, lumbar spinal stenosis, lumbar disc herniation, and thoracolumbar kyphosis. The patients underwent spine surgery with internal fixation (Figs. 1 and 2; Table 1). Three patients developed a fever after surgery between August 6 and 15, 2019, with surgery-fever intervals of 4–12 d. Despite the initial absence of fever in the fourth patient, *A. hydrophila* was isolated from their drainage fluid 6 d-post surgery. *A. hydrophila* alone was isolated from the blood or drainage fluid samples of all patients (Fig. 1), suggesting that *A. hydrophila* might have been the causative agent of the outbreak. Antibiotic treatments administered to all patients mainly included meropenem and vancomycin. The fever of the patient 1 and patient 2 subsided with the meropenem and vancomycin treatments. Despite repeated changes in antibiotic treatment for the third patient, the patient’s fever persisted and the drainage fluid cultures consistently contained *A. hydrophila*. To control the infection, a reoperation was performed to remove internal fixation and debridement, after which the patient’s temperature was eventually restored. During their stay in the hospital, the patients presented complications including cerebrospinal fluid (CSF) leak, poor wound healing, acute kidney injury, or acute heart failure. All four patients were eventually discharged, with the hospitalization duration being 18–53 d (Table 1).

### Identification of isolates and setting cultures

Matrix-assisted laser desorption/ionization time-of-flight mass spectrometry (MALDI-TOF MS) and SpeciesFinder 2.0 identified all three isolates as *A. hydrophila.* The ANI of all three strains was > 95% compared to that of *A. hydrophila* ATCC 7966, which satisfies previously established criteria for assigning the same species. In addition, the evolutionary tree of the core genes of *Aeromonas* indicated that the three clinical isolates were closely clustered with *A. hydrophila* (Fig. S2).

A total of 50 environmental samples were collected from the disinfectants, sterile cotton swabs, bone grain, infusion packs, surgical instruments, and handrails of the resident beds in five rooms. None of the samples were culture-positive. Fecal cultures for the four hospitalized patients were negative for *A. hydrophila*.

**Table 1:** Clinical records of patients in the outbreak

Characteristic	Patient 1	Patient 2	Patient 3	Patient 4
Sex	Male	Male	Female	Male
Age (years)	36	73	77	38
Admission date	July 29, 2019	July 19, 2019^*^	July 29, 2019	August 29, 2019
Diseases related to admission for spine surgery	Cervical spondylosis	Lumbar spinal stenosis, lumbar disc herniation	Lumbar spinal stenosis, lumbar disc herniation	Kyphosis
Underlying diseases	None	Hypertension, hepatitis B infection	Diabetes	Ankylosing spondylitis
Surgery date	August 2, 2019	July 26, 2019^*^	August 5, 2019	September 3, 2019
Location of surgery	Cervical	Lumbar	Lumbar	Thoracolumbar
Implants	Yes	Yes	Yes	Yes
Date of fever onset	August 6, 2019	August 7, 2019^*^	August 15, 2019	None
Days of onset of fever following surgery	Four	12	10	None
First isolation of *A. hydrophila*	August 6, 2019	August 11, 2019	August 13, 2019	September 9, 2019
Symptoms	Fever	Fever, chills	Fever, redness, and swelling of the right upper limb	None
Treatments	MRP, VAN	CAZ, MRP, VAN, ATM, LEV, CFM	FEP, MRP, VAN, LZD, LEV	MRP, VAN, LZD, CFM
Discharge date	September 4, 2019	August 26, 2019	September 20, 2019	September 16, 2019
Days of hospitalization	37	38	53	18
Complications	CSF leak, poor wound healing	CSF leak, atrial fibrillation, acute kidney injury	CSF leak, poor wound healing, acute heart failure	Implant fracture 1 year after surgery

MRP, meropenem; VAN, vancomycin; CAZ, ceftazidime; CFM, cefixime; FEP, cefepime; ATM, aztreonam; LEV, levofloxacin; LZD, linezolid; CSF, cerebrospinal fluid

^*^ The patient 2 developed a fever on July 26, 2019, the day of the spine surgery. Although ceftazidime was administered, the fever persisted for four days, and *A. hydrophila* was not detected during that period. On August 7, 2019, due to a CSF leak, the patient received puncture of subcutaneous effusion near the surgical site. On the same day, the patient developed another fever, and *A. hydrophila* was identified in the blood cultures four days later

### Sequencing and genomic analysis

We performed sequencing of the 19B23009, 19W05620, and 19W06265 genomes, and determined that their total genome sizes were all 4.88 Mb. The three de novo assemblies resulted in draft genomes composed of few scaffolds (140, 163, and 146) with high N50 values (387,668 bp, 387,667 bp, and 387,668 bp, respectively). The three draft genomes had an average GC content of 61%. Variation in the GC content of the genome is shown in the inner circle of Supplement Figure S1.

### MLST of isolates and pairwise single nucleotide polymorphisms in the core genome

**Fig. 3:**
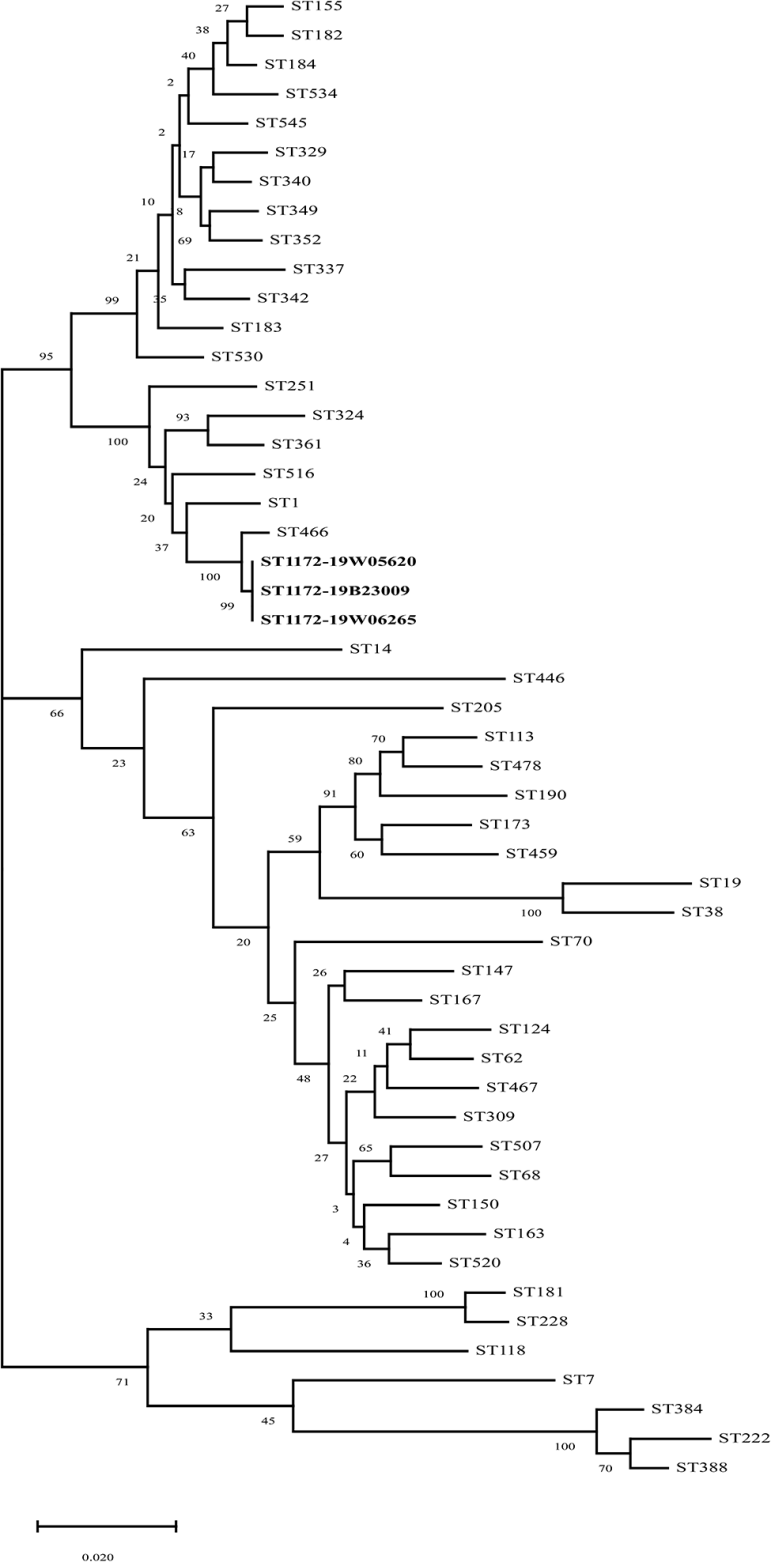
Maximum likelihood trees constructed from concatenated nucleotide sequences (*gltA*-*groL*‐*gyrB*‐*metG*‐*ppsA*‐*recA*) using MEGA X. ST1172 formed clusters with ST516 and ST251

MLST analysis of all *A. hydrophila* isolates revealed that they belonged to a novel sequence type (ST1172, deposited in the PubMLST database: https://pubmlst.org/aeromonas/). We compared the loci of housekeeping genes of ST4523 (*gyrB*, *groL*, *gltA*, *metG*, *ppsA*, and *recA*: loci 801, 331, 334, 337, 361, and 355, respectively) with ST466 (loci 338, 331, 334, 337, 361, and 355) and found that *gyrB* was mutated from sequence 1 to 2. In the phylogenetic tree, ST1172 formed clusters with ST251 and ST516 (Fig. 3). The multiple sequence alignment of strains ST251, ST516, and ST1172 is shown in Supplementary Figure S3.

### Antibiotic resistance profile and phenotypic ESBL detection

All three isolates were susceptible to amikacin and levofloxacin but resistant to imipenem, piperacillin/tazobactam, ceftriaxone, cefuroxime, and cefoxitin (Table 2). Isolate 19W05620 had increased ceftazidime resistance (minimum inhibitory concentration ≥ 64 µg/mL). All three isolates possessed the same chromosomally encoded β-lactamases, including *bla*_OXA-724_ (β-lactamase), *imiH* (metallo-β-lactamase [MBL]), and *bla*_MOX-13_ (AmpC) in plasmids (Table 3).

**Table 2:** Antimicrobial susceptibility patterns of three *A. hydrophila* isolates

Antibiotic class	Antibiotic	19B23009 (Patient 1)			19W05620 (Patient 3)			19W06265 (Patient 4)	
		MIC / Inhibition diameter (µg/mL/mm)	Interpretation		MIC / Inhibition diameter (µg/mL/mm)	Interpretation		MIC / Inhibition diameter (µg/mL/mm)	Interpretation
β-Lactams	Piperacillin/Tazobactam	≥ 128	Resistant		≥ 128	Resistant		≥ 128	Resistant
	Ceftriaxone	8	Resistant		≥ 64	Resistant		8	Resistant
	Ceftazidime	2	Susceptible		≥ 64	Resistant		2	Susceptible
	Cefepime	≤ 0.12	Susceptible		4	Intermediate		≤ 0.12	Susceptible
	Cefoperazone/Sulbactam	16	No criteria		≥ 64	No criteria		16	No criteria
	Cefuroxime	≥ 64	Resistant		≥ 64	Resistant		≥ 64	Resistant
	Cefoxitin	≥ 64	Resistant		≥ 64	Resistant		≥ 64	Resistant
	Imipenem	8	Resistant		8	Resistant		8	Resistant
	Meropenem	31	Susceptible		13	Resistant		26	Susceptible
	Amoxycillin/clavulanic acid	4	No criteria		≥ 32	No criteria		4	No criteria
Aminoglycoside	Amikacin	≤ 2	Susceptible		≤ 2	Susceptible		≤ 2	Susceptible
Trimethoprim-sulfonamide	Trimethoprim-Sulfonamide	8	No criteria		≥ 16	No criteria		8	No criteria
Fluoroquinolone	Levofloxacin	0.5	Susceptible		2	Susceptible		0.5	Susceptible
Glycylcycline	Tigecycline	≤ 0.05	No criteria		≤ 0.05	No criteria		≤ 0.05	No criteria

MIC, minimum inhibitory concentration

**Table 3:** Antimicrobial genotype prediction of the three *A. hydrophila* isolates

Antibiotic class	Resistance gene	19B23009 (Patient 1)				19W05620 (Patient 3)			19W06265 (Patient 4)	
		Match in chromosome (%)	Match in plasmid (%)			Match in chromosome (%)	Match in plasmid (%)		Match in chromosome (%)	Match in plasmid (%)
β-Lactams	*bla*_OXA-724_	98.86				98.86			98.86	
	*imiH*	96.05				96.06			96.06	
	*bla*_MOX-13_			94.52			94.52			94.52
Fluoroquinolone	*adeF*	49.23/43.71				43.71/49.23			43.71/49.23	
	*rsmA*	92.73				92.73			92.73	
Tetracycline	*Tet(C)*	100.00				100.00			100.00	
Phenicol	*CatB3*	100.00				100.00			100.00	
Aminoglycoside	*aadA*	100.00				100.00			100.00	
	*aadA16*			98.52			98.52			98.52
Sulfonamide	*sul1*			99.64			99.62			99.64
Disinfecting agents and antiseptics	*qacEdelta1*			100.00			100.00			100.00

### Distribution of virulence determinants

We screened virulence factors related to the pathogenicity of *A. hydrophila* using the VFDB and detected the toxin factors aerA/act, ahh1, ast, hlyA, hemolysin III, and thermostable hemolysin along with the T3SS secretion system and many other adherence factors, such as flgC and flaB (Supplement Table S1). The expression of the secretion system (86 items), toxin (12 items), immune evasion (1 item), serum resistance (1 item), and *lps rfb* locus (*Klebsiella*) were compared in Supplement Table S1.

## Discussion

Many *Aeromonas* species have been reported to cause infections in fish, poultry, and humans [2]. At our institution, *A. hydrophila* has been isolated from deep infections using clinical cultures in six to seven patients per year; the present study reports the first cluster of *A. hydrophila* infections within 2 months. Notably, the current investigation was based on a published report of a hospital-acquired *A. hydrophila* outbreak [3]. *Aeromonas* infections are often misdiagnosed as *Vibrio* infections before laboratory identification, which may lead to inappropriate antimicrobial administration and ineffective treatment [19]. In addition, previous studies reported the misidentification of *A. hydrophila* as *A. caviae* based on their similarity and noted the risk of *Aeromonas* misidentification when using MALDI-TOF MS alone (identification error < 10%) [20, 21]. Therefore, we evaluated ANI based on draft genomes and constructed a pan-genome tree to identify the isolated strain as *A. hydrophila.*

*A. hydrophila* mainly causes gastrointestinal diseases [2]; however, of the four isolates obtained in our study, two were isolated from sterile body fluids and two were isolated from blood. We further reviewed the clinical diagnosis and treatment of the four patients. Patients 1, 2, 3, and 4 were housed in the orthopedic ward. Despite negative culture results for both environmental and surgical instrument samples, we speculate that an outbreak of *A. hydrophila* may have occurred. We examined the clonality of the three isolates by MLST and confirmed that all the isolates were assigned to a novel ST (ST1172), which displays a unique combination of allele numbers across the six loci employed in the MLST analysis. The MLST database has been updated to include the identification of the novel allele (gyrB 801) and ST (ST1172). Based on the novel ST and the clinical presentation of the patients, we hypothesized that *A. hydrophila* may have been transmitted in the orthopedic ward.

Resistance of *A. hydrophila* to broad-spectrum cephalosporins or carbapenems has been reported, though it is uncommon in *A. hydrophila* isolates [6]. In this study, all three isolates exhibited resistance to imipenem, piperacillin/tazobactam sodium, ceftriaxone, ceftazidime, cefuroxime sodium, and cefoxitin. Three patients had severe spinal infections with fever; meropenem was administered since imipenem is contraindicated for increased risk of neurological infection. Therefore, the emergence of this multidrug-resistant *A. hydrophila* may have an impact on therapeutic decisions.

Despite previous studies showing that *A. hydrophila* naturally encodes class B MBLs as well as CphA and class D-β-lactamases, only *imiH*, *bla*_MOX-13_, and *bla*_OXA-724_ were detected in our study. The *imiH* class B MBLs are unique carbapenemases whose active sites require zinones, and they are widely distributed in clinical and environmental strains [22]. The class C β-lactamase (AmpC) *bla*_MOX-13_ was present in a plasmid, and was previously detected in environmental strains [22]. Notably, in patient 3, the genome of the meropenem-resistant isolate (19W05620) was indistinguishable from genomes 19B23009 and 19W06265 regarding resistance genes. β-Lactamase and outer membrane protein mutations in 19W05620 did not elucidate the resistance mechanism. Therefore, we hypothesized that the resistance of 19W05620 may be attributed to the overexpression of β-lactamase or efflux pump systems.

Analysis of the virulence genes revealed that the virulence factors aerA/act, ahh1, ast, hlyA, hemolysin III, and thermostable hemolysin were present in all three isolates. We also found that all isolates had type III and VI secretion system genes. Sierra et al. reported that with the type III secretion system, toxins can be inserted into host cells [23], and Bingle et al. reported that virulence factors can be inserted into host cells using valine-glycine repeat proteins and hemolysin-coregulated proteins via type VI secretion system [24]. Thus, the virulence factors of *A. hydrophila* may prolong the duration of patient hospitalization and affect prognosis.

*A. hydrophila* was not isolated from the environment or equipment, and no further cases of *A. hydrophila* infection were reported in the ward after complete cleaning and disinfection. The main limitations of this study were the analysis of only three isolates and selection of the first isolate alone from each patient. We were also unable to explore whether strain resistance to carbapenem was related to antibiotic usage. In addition, we were unable to determine a primary origin of this organism since results of environmental screening were negative.

## Conclusions

This study reports the outbreak of carbapenem-resistant *A. hydrophila* ST1172 in an orthopedic ward and emphasizes the need for improved control measures to prevent further dissemination of such organisms in hospital settings. Furthermore, there have been several reports of carbapenemase-producing Aeromonas strains causing nosocomial infections [5]. Hence, it is crucial to give due consideration to the potential risk of transmitting multidrug-resistant *A. hydrophila*

### Electronic supplementary material

Below is the link to the electronic supplementary material.

**Supplementary Material 1: Supplement Table S1.** Summary of virulence factor genes in *Aeromonas hydrophila* isolates obtained via comparison of protein sequences in the VFDB

**Supplementary Material 2: Supplement Figure S2.** Phylogenetic tree constructed based on core genes identified and aligned using Roary software

**Supplementary Material 3: Supplement Figure S3.** Complete sequence alignments of ST251, ST516, and ST1172

**Supplementary Material 4: Supplement Figure S4.** Comparison of 19B23009 genome with 19W05620 and 19W06265. Starting from the outer ring and moving inward, the six green circles represent open reading frames (ORFs) and the next circle displays the GC content and GC skew of the reference sequence. Blast comparisons with other strains are depicted in the outermost circle

## Data Availability

All data generated or analyzed during this study are included in this article.

## List of abbreviations

MLST: Multilocus sequence typing
CSF: Cerebrospinal fluid
AmpC: Ambler class C
ESBL: xtended-spectrum-?-lactamase
ANI: Average nucleotide identity
MIC: Minimum inhibitory concentration
WGS: Whole genome sequencing
MALDI-TOF MS: Matrix-assisted laser desorption/ionization time-of-flight mass spectrometry
