# Supplementary figures and images for: Emergence and clonal expansion of Aeromonas hydrophila ST1172 that simultaneously produces MOX-13 and OXA-724
Source: Antimicrob Resist Infect Control. 2024 Mar 3;13:28. doi: 10.1186/s13756-023-01339-4 (PMC10910732; doi:10.1186/s13756-023-01339-4)

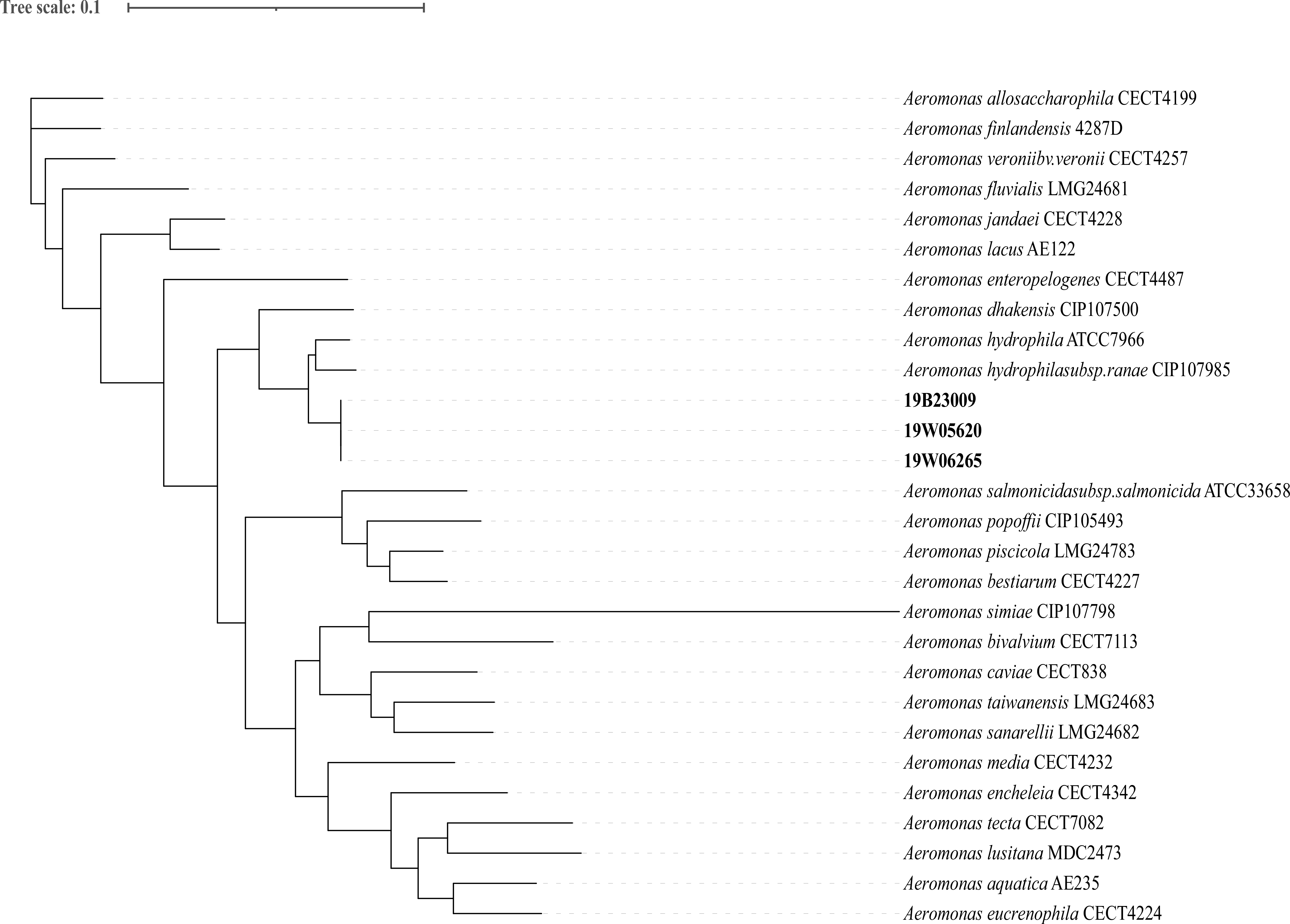

Supplement: Supplementary file 2 — Supplementary Material 2: Supplement Figure S2. Phylogenetic tree constructed based on core genes identified and aligned using Roary software [file 13756_2023_1339_MOESM2_ESM.tif]

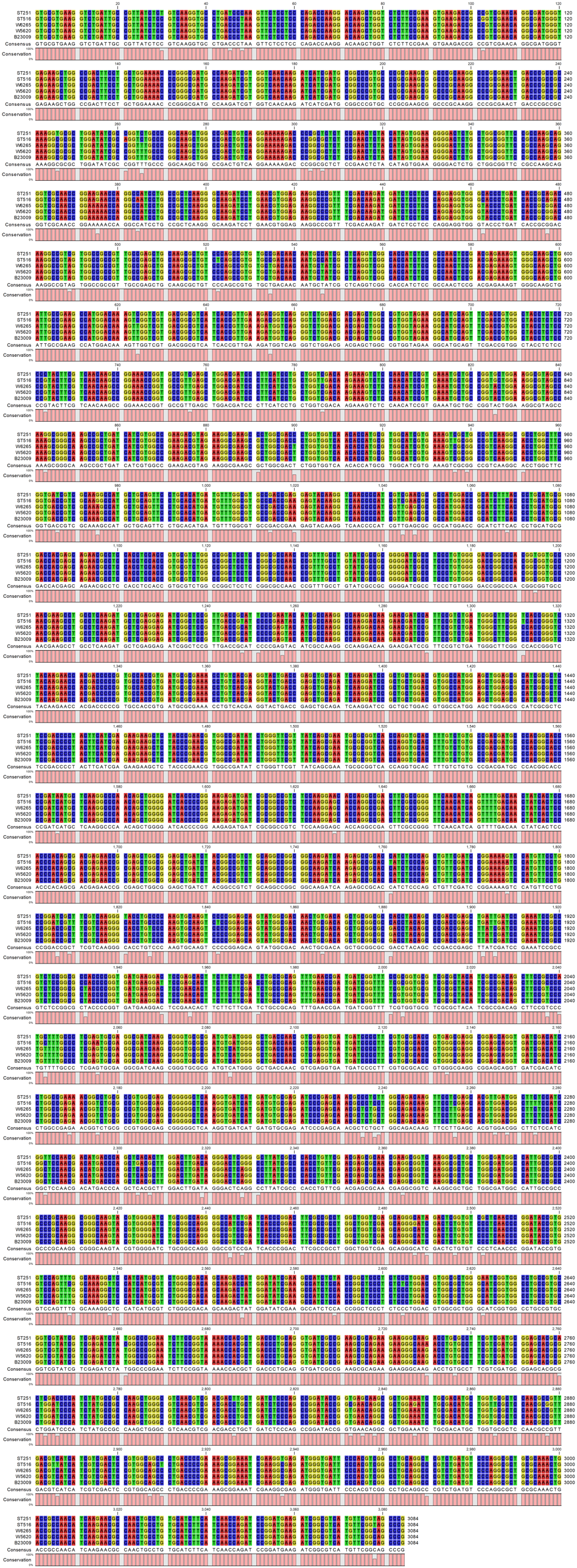

Supplement: Supplementary file 3 — Supplementary Material 3: Supplement Figure S3. Complete sequence alignments of ST251, ST516, and ST1172 [file 13756_2023_1339_MOESM3_ESM.tif]

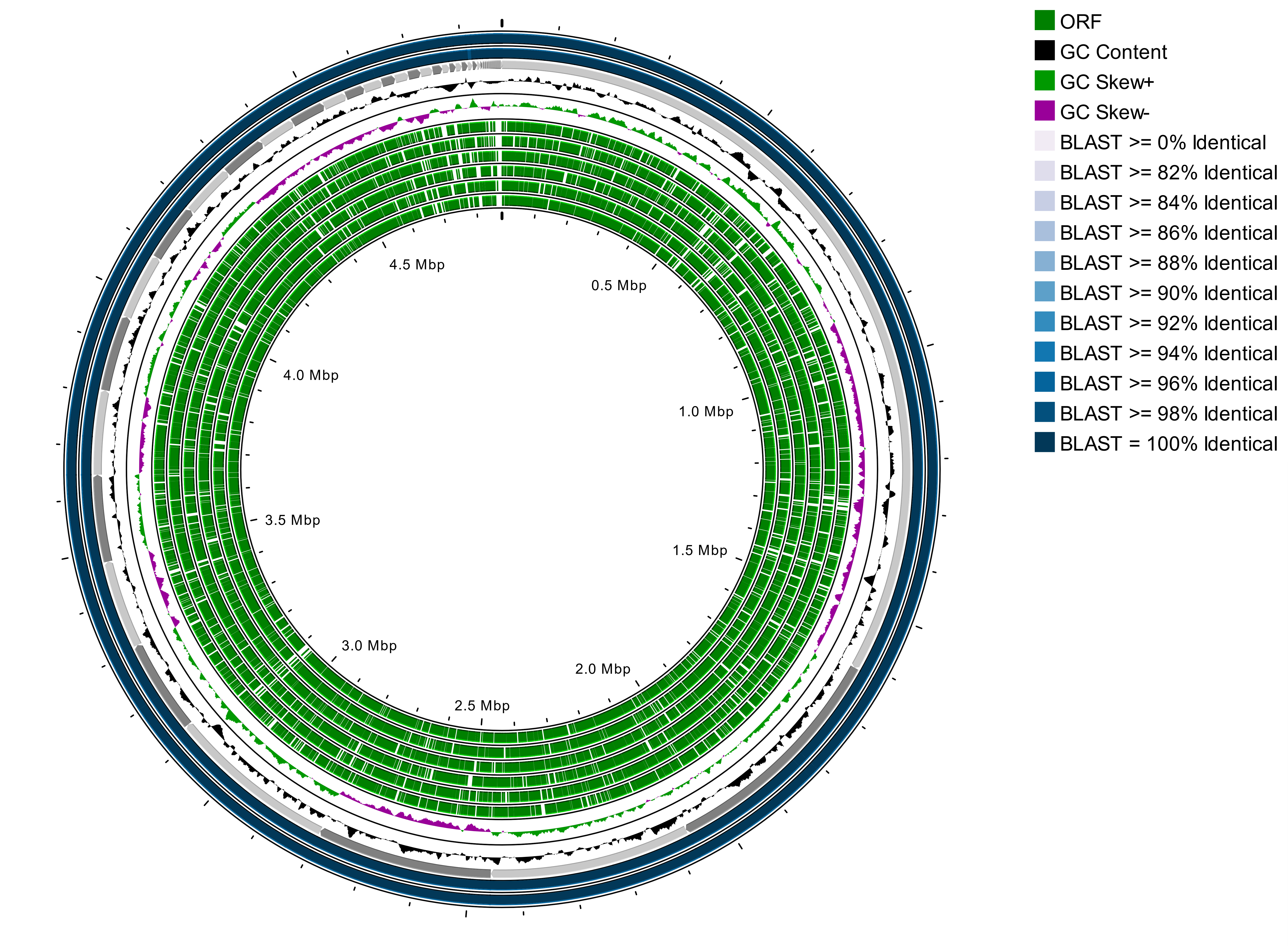

Supplement: Supplementary file 4 — Supplementary Material 4: Supplement Figure S4. Comparison of 19B23009 genome with 19W05620 and 19W06265. Starting from the outer ring and moving inward, the six green circles represent open reading frames (ORFs) and the next circle displays the GC content and GC skew of the reference sequence. Blast comparisons with other strains are depicted in the outermost circle [file 13756_2023_1339_MOESM4_ESM.tif]
